# Private-sector investor’s intention and motivation to invest in Land Degradation Neutrality

**DOI:** 10.1371/journal.pone.0208813

**Published:** 2018-12-13

**Authors:** Tony Reyhanloo, Stefan Baumgärtner, Matthias Haeni, Simone Quatrini, Philippe Saner, Eike von Lindern

**Affiliations:** 1 Department of Evolutionary Biology and Environmental Studies, University of Zurich, Zürich, Switzerland; 2 Department of Environmental Economics and Resource Management, University of Freiburg, Freiburg, Germany; 3 Swiss Federal Research Institute WSL, Birmensdorf, Switzerland; 4 Datascientist.ch, Wallisellen, Switzerland; 5 Department of Environmental Systems Science, Swiss Federal Institute of Technology (ETH), Zürich, Switzerland; 6 URPP Global Change and Biodiversity, University of Zurich, Zürich, Switzerland; 7 Dialog N – Research and Communication for People, Environment and Nature, Uster, Switzerland; TNO, NETHERLANDS

## Abstract

Private-sector investors could be key players in combatting global land degradation and realising the emerging concept of Land Degradation Neutrality (LDN). To better understand how to incentivize private-sector investors for LDN, we conducted an online-survey of 68 private-sector investors. Structural equation modelling based on the theory of planned behavior was performed to investigate how cognitive, social, emotional, motivational and financial determinants influence their intention and motivation to invest in LDN. Good knowledge and a positive attitude towards both LDN and investing sustainably were found to be main predictors for intention. In contrast, perceived social pressure had little effect on the intention to invest towards combating land degradation. The general motivation to invest sustainably was mainly triggered by a consciousness for sustainability and emotional attachment, less by the desire for short-term profit maximisation whilst prospects of long-term financial return are important. Overall, strong homogeneity in psychological determinants was found for both traditional and impact investors. As the determinants of the intention and the motivation to invest sustainably do not substantially differ across different investor types, our study implies that investors should be targeted as a uniform group when mobilising interest for LDN. Emphasis should be placed on the psychological determinants traditional and impact investors commonly share, rather than on the type-specific characteristics that may distinguish different investor types.

## Introduction

Land degradation, defined by the United Nations Convention to Combat Desertification (UNCCD) as the “reduction or loss […] of the biological or economic productivity and complexity [of land] resulting from land uses”, is heavily influenced by social and market-driven activities [1, 2]. Its direct effects and ramifications might eventually be felt by everyone, due to cross-scale globalisation processes, including both on a direct (e.g. loss of productivity and biodiversity, depletion of natural resources, reduced agricultural commodities, increase in prices, market failures) and an indirect level (e.g. higher temperature increases, reduced resilience to climate change, drought, hunger, conflicts, migrations [3,4,5].

The United Nations recognized that land degradation is a major global challenge and renewed their commitment to tackle it by adopting a distinct target under Sustainable Development Goal n°15: Life on Land [6]. Target 15.3 specifically engages the international community “by 2030, to combat desertification, restore degraded land and soil, including land affected by desertification, drought and floods, and strive to achieve a land degradation-neutral world” [6]. The concept of Land Degradation Neutrality (LDN) envisions "a world where the amount of healthy and productive land resources necessary to support ecosystem functions and services and enhance food security remains stable or increases within specified temporal and spatial scales and ecosystems" [2].

Achieving LDN hinges upon the implementation of sustainable land management practices and the ecological restoration of degraded lands and abandoned rural lands [2]. This two-pronged strategy will have to be deployed worldwide, using an integrated landscape approach, in order to reverse the current land degradation trend, which is estimated to cause an annual loss of 12 million hectares of land, 15 billion trees and 24 billion tons of fertile soil resources [7]. Undoubtedly, this will imply high costs, which however would prevent much higher costs in terms of losses in land-based ecosystem services, estimated to about USD 6.3–10.6 trillion per year, or 10–17% of the global GDP depending on different estimates [8,9]. Hence, in economic terms, implementing LDN is an investment, which requires large amounts of financial capital upfront and might offer long-term financial returns to investors.

Also, the Intergovernmental Science-Policy Platform on Biodiversity and Ecosystem Services (IPBES) confirms that land degradation is a global, pervasive threat to biodiversity and ecosystem services [10]. It urges the international community and private sector to undertake a concerted response against land degradation and invest in avoidance, mitigation and restoration strategies. It underlines the importance of internalizing a broader range of factors in investment decisions beyond short-term financial returns, such as environmental, social and non-market costs and benefits. This would require a ‘step change’ in education and awareness-raising efforts to inform perceptions and values that guide investment decisions.

This raises the question of which sources of capital can be mobilised for LDN, and how. The vast majority of LDN-related activities to date implemented under the UNCCD have been financed by the public sector [11]. Despite evidence of wide success [12] and multiple benefits officially reported from such measures [13], investments in LDN remain marginal and limited [11]. Given the limitations of the public sector to provide sufficient financial resources to address problems associated with land degradation, it is becoming increasingly clear that the crucial challenge ahead for LDN is to mobilise the required financial capital from the private sector.

Our study seeks to better understand the multifaceted determinants and barriers of private sector investments in LDN, e.g. investments that promote land restoration in general and sustainable land management practices. Examples of LDN investments include reforestation of degraded forestland in accordance with the highest standards for sustainable forest management (such as Forest Stewardship Council), or productive landscape restoration projects in degraded areas via agroforestry schemes [12]. The main incentive for a private-sector investor would be that the restoration of degraded land itself returns a financial profit, as the land is worth more after the restoration than before [14]. Paetzold and Busch’s study (2014) of individual private wealth investors found that investors associated sustainable investments with high volatility, financial losses and a short investment time horizon that prevent them from investing sustainably [15]. This discrepancy between investor interest and actual engagement in investing sustainably is known as the ‘sustainable investing gap’ [15]. Specifically, within the frame of investing in natural resources (incl. land), investors seem to perceive sustainable investments as alternative and risky as well as leading to low financial returns, which tend be generated over a longer time horizon, and therefore not coinciding with other profitable short-term investment opportunities [11,16,17].

Against this background, we aimed to 1) investigate how cognitive, social, emotional, motivational and financial determinants influence private-sector investors’ intention to invest in LDN, 2) create investor profiles based on underlying motivational structures, 3) and develop practical suggestions for how to best encourage private-sector investors to invest in LDN and contribute to fulfilling the Sustainable Development Goals.

## Methods

### Questionnaire

We developed an online questionnaire in English based on the theory of planned behavior [18,19]. The questionnaire consisted of 41 items and completion took approximately 10–15 min (see S1 File). An introductory letter informed potential participants about land degradation and the LDN concept and expressed our interest to collect responses from private investors (see S1 File). Participants gave their informed consent to participate in the study implicitly by clicking the link to the questionnaire. We recorded the date and time of clicking the questionnaire link and report the birth year of each participant (no minors participated in the study). The questionnaire comprised two main parts 1) intention to invest in LDN and 2) motives for investing sustainably. Based on the “Checklist to Self-Assess Studies Concerning Their Ethical Safety” of the Ethics Committee at University of Zurich and in agreement with the faculty representative in the University Ethics Board a formal ethical evaluation of this study was not required.

### Data collection

We pre-tested the questionnaire with a set of independent experts to ensure that the answer format, scales and items were technically well-suited to answer our research questions. The questionnaire was put online between 12.04.2016–12.07.2016 (91 days). Participants were ensured anonymity and no commercial use of data that would only be processed for scientific purposes and would never be given to any third party. No incentives were offered, except for a report on the main findings of the study if the participant would wish for it. Recruitment of participants was done through the authors’ networks mainly by mail, but also in person by directly approaching an open audience of investors at several events and conferences related to both traditional and sustainable finance. Furthermore, we published the study invitation on two finance platforms, the website of the University of Zurich and LinkedIn.

### Survey target group: Private-sector investors

Our study targeted investors from the private sector. Assuming that investors can be grouped according to their investment strategy and motivation, we asked participants whether they identified themselves as traditional investors ("I make investments primarily to generate financial returns."), impact investors ("I make investments to generate financial returns that include having a positive social and/or environmental impact.") or philanthropic investors ("I make investments primarily to generate a positive social and/or environmental impact and less/no financial returns for myself." A total of 79 investors responded, including 32 traditional investors (47%), 28 impact investors (41%) and 8 philanthropic investors (12%). As the distribution of responses among these three investor types was strongly asymmetric, a revised distinction of investor type was used subsequently: we distinguished between traditional ("I make investments primarily to generate financial returns.") and impact investors ("I make investments to generate financial returns that include having a positive social and/or environmental impact."), in which the new impact investor type consists of former impact and philanthropic investors. This re-grouping seemed reasonable as both impact and philanthropic investors overlap in their aim to generate a social and/or environmental impact with their investments, which differs from the mainly profit-centred aim of traditional investors. 11 participants were excluded due to incomplete responses. Otherwise no exclusion criteria were applied, for example in terms of investors’ wealth or general volume of investments, during the participant selection. 68 complete questionnaires were obtained, including 32 from traditional investors (47%) and 36 from impact investors (53%), resulting in an almost 50:50 ratio. Half of the respondents had natural resources (incl. land) as part of their investment strategy while the other half did not. The majority of investors in our sample was male (81%), from Switzerland (62%), with a mean age of 47 years (standard deviation = 13.4 years). 74% of investors stated to be independent in their investment decision-making. No accurate response rate can be calculated, as it is unclear how many participants were initially reached. As socio-demographic and investor-based data on the population of all private-sector investors was not available to us, we could not check whether our sample is representative of private-sector investors or not.

### Data analysis and modelling

To study the intention to invest, we applied the theory of planned behavior (TPB), which is a parsimonious social-cognitive model, as previously used in both sustainable behavior studies [20] and sustainable finance [15]. The TPB posits that behavior is determined by an intention to perform the behavior, which in turn is formed by three antecedent determinants: 1) attitude towards the behavior, 2) subjective norm (= perceived social pressure) and 3) having the perceived behavioral control of performing the behavior. The greater and more favourable these variables are, the higher is the likelihood that an intention is formed and thus behavior performed. Due to lack of data and the relative novelty of LDN as an investment opportunity we could not integrate actual investments in LDN into the analysis and therefore used investors’ self-reported intention to invest in LDN in 2016/2017. Modified to the context of this study, the intention is predicted by investors’ 1) attitude towards investing in LDN, 2) subjective norm, 3) knowledge about LDN and investment vehicles in LDN as well as 4) the ability and financial resources to invest in LDN (see Table 1). Participants rated statements based on these constructs with 6-point Likert-scales indicating levels of agreement (6 = *strongly agree*, 5 = *agree*, 4 = *somewhat agree*, 3 = *somewhat disagree*, 2 = *disagree*, 1 = *strongly disagree*). Attitude consisted of investors’ overall positive or negative evaluation of LDN being an effective solution to combat land degradation and attractive investment opportunity that will yield both high financial return and positive impact. Subjective norm reflects investors’ perception of being socially pressured by people from their working life, their private life and the public opinion (e.g. media) to be concerned about land degradation and therefore to invest in LDN. We regarded the construct perceived behavioral control of intending to invest in LDN as two-fold, which we separated in 1) having knowledge about LDN and investing sustainably in LDN as well as 2) having the ability and financial resources to invest. In addition to the constructs of the TPB, we added three constructs that linked personal connection of investors to the issue of land degradation (will be discussed in more detail below): 1) perceived victim of land degradation, 2) perceived contributor to land degradation as well as 3) closeness to the asset class of natural resources (including land).

**Table 1:** Overview of items used for the intention to invest in LDN analysis.

Construct		Item	Response format
**Attitude towards investing in LDN**	**Attitude 1**	*"To the best of my knowledge*, *I think Land Degradation Neutrality is a promising solution to counteract land degradation in the long-term*.*"*	Likert scale (6 = *strongly agree*, 5 = *agree*, 4 = *somewhat agree*, 3 = *somewhat disagree*, 2 = *disagree*, 1 = *strongly disagree*)
	**Attitude 2**	*"I think sustainable investment products based on the concept of Land Degradation Neutrality could be an attractive investment opportunity*.*"*	
	**Attitude 3**	*"Generally*, *I am confident that investing in sustainable investment products will yield a high financial return and have a positive impact*.*"*	
	**Attitude 4**	*"I am convinced that sustainable investment products promote sustainable development in an effective way*."	
**Subjective norm**	**Subjective norm 1**	*"The people in my working life*, *whose opinion I value*, *expect me to invest in Land Degradation Neutrality in an effort to combat LD*.*"*	
	**Subjective norm 2**	*"The people in my private life*, *whose opinion I value*, *expect me to invest in Land Degradation Neutrality in an effort to combat LD*."	
	**Subjective norm 3**	*"There is strong public concern about land degradation as a serious threat*, *which is one of the reasons I should make investments in Land Degradation Neutrality in an effort to combat it*.*"*	
**Knowledge about LDN/sustainable investing**		*"I am NOT in a position to invest in Land Degradation Neutrality because I do NOT know how to do so*.*"*	
**Ability and financial resources**		*"I have the ability/financial resources to invest in sustainable development AND/OR Land Degradation Neutrality*.*"*	
**Intention of investing in LDN (in 2017)**		*"I will invest/increase my investments in Land Degradation Neutrality in 2017 to make a contribution to counteracting land degradation*.*"*	
**Perceived victim of land degradation**		*"I am directly affected by the negative effects of land degradation*.*"*	Yes, No, Maybe
**Perceived contributor to land degradation**		*"My previous investment decisions may have directly contributed to generating land degradation*.*"*	Yes, No, Maybe
**Closeness to asset class**		*"Are natural resources (incl*. *’land’) as an asset class part of your investment strategy*?*"*	Yes, No, Maybe

We performed structural equation modelling (SEM) for analysing the intention to invest, using the R package *lavaan* [21]. As a first step, we formulated the measurement model to test with simultaneous confirmatory factor analyses whether the latent variables were represented well by the manifest indicators. Additionally, we conducted scale reliability analyses by calculating Cronbach’s α, testing internal consistencies and item-total correlations. Cronbach’s α indicated acceptable internal consistency for attitude (α = 0.74) and good internal consistency for subjective norm (α = 0.87). Item-total correlations of attitude (Attitude 1 = 0.40, Attitude 2 = 0.59, Attitude 3 = 0.58, Attitude 4 = 0.50) and subjective norm (Subjective Norm 1 = 0.80, Subjective Norm 2 = 0.81, Subjective Norm 3 = 0.68) were satisfactory. Therefore, no items needed to be dropped from subsequent analysis.

**Fig 1:**
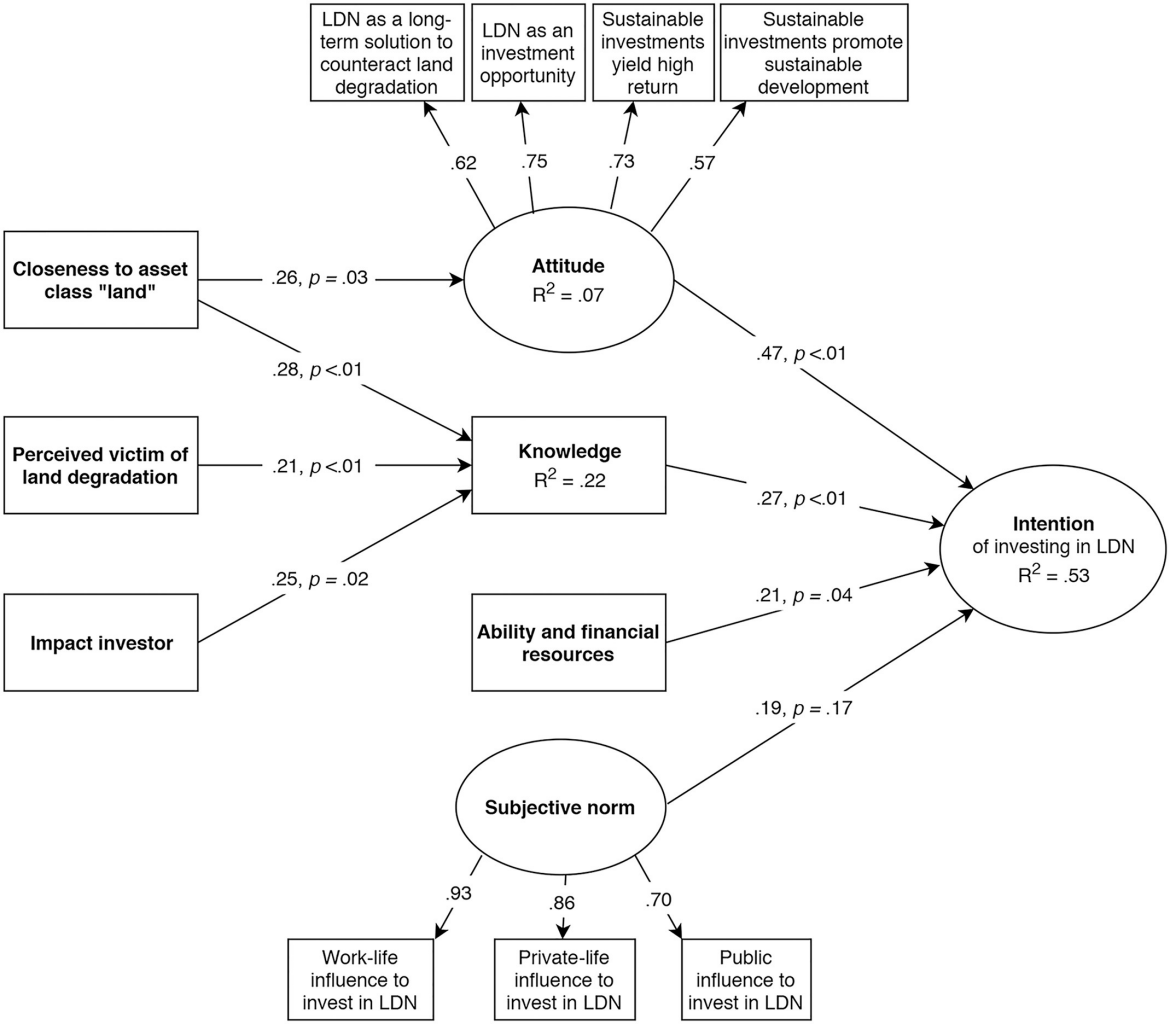
Structural equation model of how the intention to invest in LDN can be explained based on the theory of planned behavior, with standardised coefficients (betas) and *p*-values for all predictors of intention as well as standardised factor loadings of manifest variables on their corresponding latent variables (see Tables 1 and 2 for details). Round boxes represent latent constructs, consisting of multiple items, while square boxes are manifest constructs, captured by one single item.

Next, we included the regression paths postulated by TPB, before finally defining regression paths from latent variables associated with LDN to TPB constructs (see Fig 1 for the complete model). Following this stepwise procedure, we ensured that the model evolved according to theoretical consideration. To account for possible non-normality in the underlying data, we calculated robust standard errors. To evaluate the SEM fit, the chi-square value divided by degrees of freedom (Chi-square/df), comparative fit index (CFI), Tucker-Lewis index (TLI), root mean squared error of approximation (RMSEA) and standardized root mean squared residual (SRMR) were used [22,23]. We consider CFI and TLI values close to or greater than 0.95, the SRMR and RMSEA close to or below 0.08 and 0.06, respectively, as well as a Chi-square/df value between 1 to 3 as indicating an acceptable model fit [22,24]. Yet, what fit indices in which combination represent an acceptable model fit is an ongoing controversy [24,25]. This means that the values stated above represent no strict cut-off criteria, but rather desirable values.

Furthermore, we incorporated into the model socio-demographic and investor-based variables (traditional vs. impact investors, individual vs. institutional investors, gender, different age groups), to test whether specific investor types directly or indirectly allowed predicting the intention to invest. Moreover, we added three additional constructs to the model: 1) closeness to asset class ‘land’, 2) being a perceived victim of land degradation, and 3) being a perceived contributor to land degradation, all of which reflect personal connections investors have to land degradation/LDN as well as land-related and sustainable investments. We hypothesised that these constructs influence the intention to invest in LDN positively. Closeness was defined by whether investors had natural resources (incl. land) as part of their investment strategy (i.e. past investments in land-based investment products) and are thus familiar with this investment domain. Gaining closeness through past experience/investments may be crucial, as investors perceive investment products to be safer the more easily understandable they are [26], which could have positive effects on attitude-behavior as well as intention-behavior relationships. To increase explanatory power, we also conceptualised the constructs of investors perceiving themselves as being negatively affected by the adverse effects of land degradation on their businesses ("victims") as well as perceiving themselves as contributors to land degradation by previously made investments that may have caused land degradation.

To study the motivation for sustainable investing, a set of 19 motives was identified (by expert interviews and literature research), which were a priori structured in five higher motive-categories—wealth gain (i.e. financial motives), social influence, identity/trait (residual group), emotional attachment and sustainability consciousness (i.e. ethical and moral motives)–and confirmed through reliability analyses (see S1 Table for definitions). Participants rated on a 7-point Likert-scale to which degree they found each of the 19 motives to be influential (1 = not influential at all, 7 = very influential) when making sustainable investments. As for intentions, we expected motives to differ for traditional and impact investors, which we tested with Wilcoxon rank sum tests. In addition to the test statistics (*W* and *p*-values), we also provide Cohen’s *d* as measure for effect size. A Cohen’s *d* of 0.2 is considered a small effect, *d* = 0.5 a medium effect, and *d* = 0.8 (or higher) a large effect. See e.g., [27] for details on Wilcoxon rank sum test or Cohen’s *d*.

## Results

Surveyed investors generally had an overall positive attitude towards LDN and found potential LDN-based investment products attractive (mean = 4.55, median = 5, standard deviation = 0.75) (see Table 2).

**Table 2:** Correlation matrix and descriptive results of TPB-based variables for the intention to invest in LDN analysis.

Construct	Item	Mean (Standard Deviation)	Standard Error	Median	Correlations matrix									
					LDN as a long-term solution to counteract land degradation	LDN as an investment opportunity	Sustainable investments yield high return	Sustainable investments promote sustainable development	Work-life influence to invest in LDN	Private-life influence to invest in LDN	Public influence to invest in LDN	Know-ledge	Ability and financial resources	Intention
Attitude	LDN as a long-term solution to counteract land degradation	4.85 (0.82)	0.10	5										
	LDN as an investment opportunity	4.37 (1.01)	0.12	4	0.49**									
	Sustainable investments yield high return	4.34 (1.13)	0.14	4	0.27*	0.55**								
	Sustainable investments promote sustainable development	4.63 (0.96)	0.12	5	0.29*	0.47**	0.63**							
Subjective norm	Work-life influence to invest in LDN	3.10 (1.33)	0.16	3	0.41**	0.43**	0.44**	0.38**						
	Private-life influence to invest in LDN	3.43 (1.36)	0.17	4	0.37**	0.32**	0.32**	0.28*	0.80**					
	Public influence to invest in LDN	3.56 (1.19)	0.14	4	0.30*	0.14	0.30*	0.25*	0.63**	0.65**				
Knowledge		2.99 (1.52)	0.18	3	0.25*	0.21	0.21	0.26*	0.28*	0.15	0.11			
Ability and financial resources		4.01 (1.34)	0.16	4	0.23	0.53**	0.31**	0.29*	0.26*	0.24*	0.11	0.20		
Intention		3.34 (1.25)	0.15	4	0.44**	0.57**	0.51**	0.41**	0.55**	0.42**	0.35**	0.45**	0.48**	

(N = 68, * = *p* < 0.05, ** = *p* < 0.01, Likert-scale range from ‘*strongly agree* (6)’ to *strongly disagree* (1)’, M = Mean, *SD* = Standard deviation, SE = Standard error).

The overall positive attitude also reflected that investors perceived LDN-based investments to yield both high financial returns alongside having a positive impact on sustainable development. The perceived social influence from working and private life and public concern (i.e. a subjective norm) was a rather weak reason for investing in LDN (mean = 3.36, median = 3, standard deviation = 1.16). Surveyed investors reported a lack of knowledge about how to invest in LDN as a reason that prevents them from investing in land degradation (mean = 3.01, median = 3, standard deviation = 1.52), while at the same time having the ability and sufficient financial resources to invest in LDN (mean = 4.01, median = 4, standard deviation = 1.34). Ultimately, 50% of surveyed investors stated that they would make investments in LDN in 2016/2017 (mean = 3.39, median = 4, standard deviation = 1.25).

Overall, correlations of TPB-based variables ranged from weak to moderate relationships (see Table 2). Moderate correlations were mostly found in items (e.g. SN1-SN3) belonging to their correspondent latent construct (e.g. subjective norm). Correlations between the dependent variable (behavioral intention) and all independent variables (TPB-based items) ranged between *r* = 0.35–0.57 and were all positive (*p* < 0.01).

Almost half of the participants stated that natural resources (incl. land) was part of their investment strategy (N = 33). Investors who had not invested in natural resources (incl. land) in the past (N = 18) mostly claimed that they did not do so for ‘no specific reasons’ or reported ‘high uncertainty’, ‘low expected return’ and ‘ethical considerations and doubts in regard to true sustainability in the asset class of natural resources in general’.

Most investors perceived themselves as not being victims of land degradation. However, about 30% of investors were undecided whether they were affected by the negative effects of land degradation or not. This revealed both strong dissociation from land degradation and high uncertainty about the issue. The majority of investors claimed to be no contributors to land degradation by past investment decisions that may had directly generated land degradation (40%). Likewise, there was an almost equally high uncertainty among the investors of being a contributor to land degradation or not (46%). Since at least half of surveyed investors reported natural resources (incl. land) as part of their investment strategy (N = 33), we further investigated how they perceived their personal connection to land degradation and found the almost exact same patterns in terms of uncertainty and dissociation about their connection to land degradation as with all investors.

Based on the TPB and the data at hand, we were able to construct a SEM (see Fig 1) of how the intention to invest in LDN is formed. In the SEM, the intention to invest in LDN was strongly predicted by attitude (beta = 0.47, *p* < 0.01) and moderately predicted by knowledge (beta = 0.26, *p* < 0.01) and ability and financial resources (beta = 0.21, *p* < 0.04). However, subjective norm did not predict intention (beta = 0.19, *p* = 0.17). Standardised factor loadings of manifest variables on their corresponding latent variables were satisfactory for attitude 0.62 (Attitude 1), 0.75 (Attitude 2), 0.73 (Attitude 3), 0.57 (Attitude 4) and for subjective norm 0.93 (Subjective norm 1), 0.86 (Subjective norm 2) and 0.70 (Subjective norm 3) (see Table 2). Though slightly lower than desired values, the model fit of the SEM can be considered satisfactory (χ^2^ /df = 1.42 (*p* < .05), Comparative Fit Index = .92, Tucker Lewis Index = .90, Root Mean Square Error of Approximation = .08, Standardised Root Mean Square Residual = .14) same with the SEM accounting for 53% of variance in intention towards investing in LDN.

Intention was neither directly predicted by the type of investor (i.e. traditional or impact) nor by socio-demographic variables or personal connection constructs. However, intention was indirectly influenced by three moderate effects on knowledge and one moderate effect on attitude: Knowledge was positively affected by impact investors (vs. traditional investors; beta = 0.25, *p* < 0.02), closeness-investors (beta = 0.21, *p* < 0.01) and victim-investors (beta = 0.21, *p* < 0.01). All three variables accounted for 22% of variance in knowledge. Therefore, when analysed with knowledge as mediator variable, small effects of impact investors (beta = 0.08, *p* < 0.02), closeness-investors (beta = 0.06, *p* < 0.01) and victim-investors (beta = 0.07, *p* < 0.01) were found on intention. Moreover, closeness-investors had a positive relationship with attitude (beta = 0.26, *p* < 0.03), which ultimately accounted for 7% of variance in attitude. As a result, the indirect effect of closeness-investors (beta = 0.12, *p* = 0.03) on intention was small when analysed with attitude as mediator variable.

**Fig 2:**
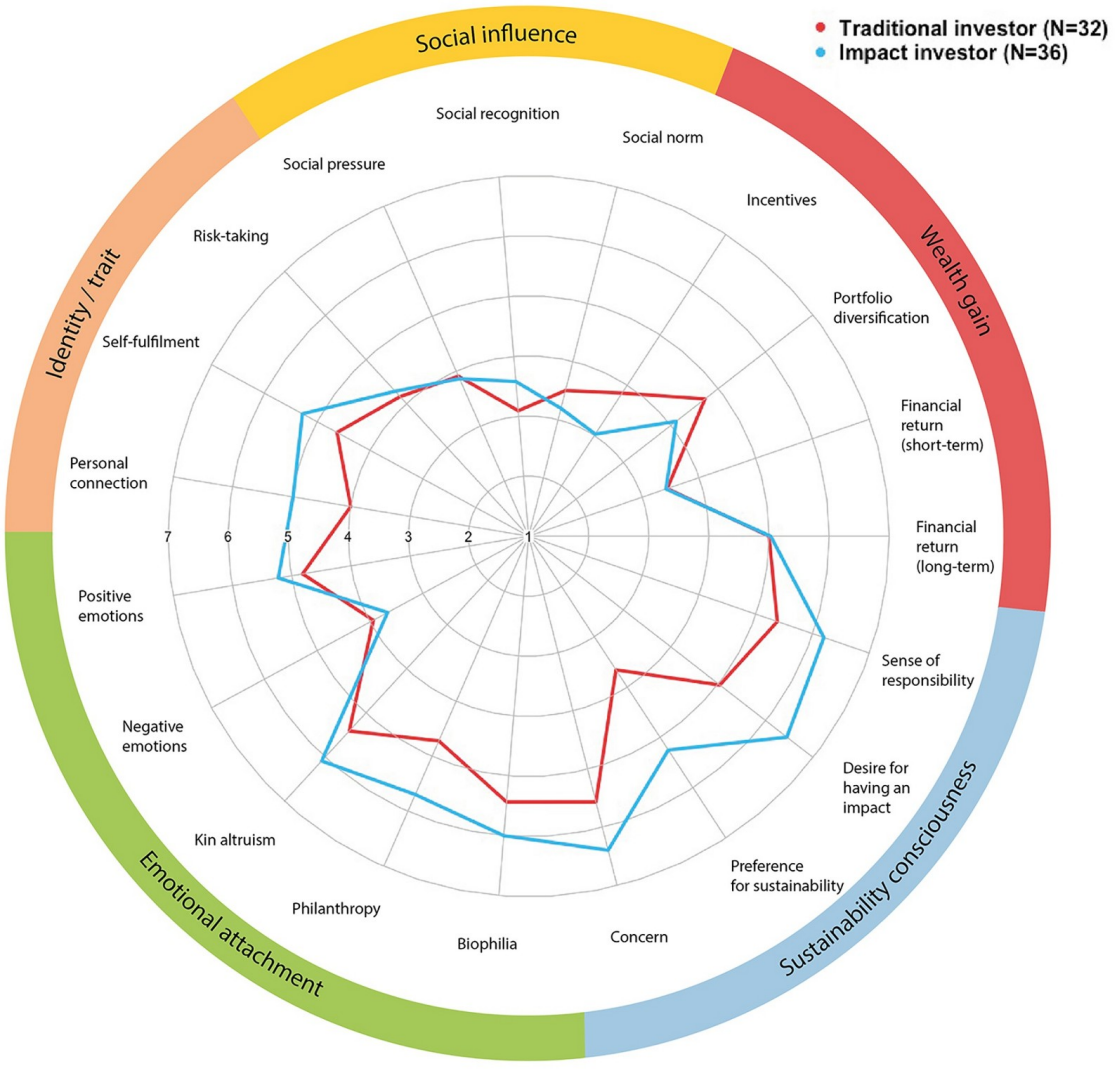
Influences of different motives on making sustainable investments by investor type (1 = motive not influential at all, 7 = very influential motive; red = mean response from all (N = 32) traditional investors, blue = mean response from all (N = 36) impact investors).

For the average investor (N = 68), the six most influential motives for making sustainable investments were ‘concern about the future’, ‘sense of responsibility’, ‘desire for having an impact’, ‘kin altruism (helping loved ones)’, ‘biophilia (love for nature)’ and ‘philanthropy (love for people)’ (means between 5–6). Closely followed by motives ‘positive emotions (happiness)’, ‘high financial return (long-term)’ and ‘self-fulfilment’ (means around 5). Therefore, the motive-categories emotional attachment and sustainability consciousness were most influential for sustainable investor behavior. In contrast, the six least influential motives for investing sustainably were ‘social norm (affiliation to trend/growing market)’, ‘social recognition (benefits image and reputation)’, ‘incentives (subsidies, tax benefits)’, ‘high financial return (short-term)’, ‘negative emotions (guilt about wrongdoings)’ and ‘social pressure’ (means below 4). Correlations of motives were mostly weak or moderate—with few high exceptions—ranging between *r* = 0.01–0.73 (see Table 3). Stronger correlations were found between motives that aggregate together to their corresponding motive-category. No significant correlations were found that indicated negative relationships among motives (*p* > 0.05). Overall, we found high variability among motives for investing sustainably (see Fig 2).

**Table 3:** Correlation matrix of motives to invest sustainably analysis.

Motive category	Motive	Correlation matrix																		
		Financial return (long-term)	Financial return (short-term)	Portfolio diversification	Incentives	Social norm	Social recognition	Social pressure	Risk-taking	Self-fulfilment	Personal connection	Positive emotions	Negative emotions	Kin altruism	Philanthropy	Biophilia	Concern	Preference for sustainability	Desire for having an impact	Sense of responsibility
**Wealth gain**	Financial return (long-term)																			
	Financial return (short-term)	0.49**																		
	Portfolio diversification	0.25*	0.22																	
	Incentives	0.37**	0.36**	0.29*																
**Social influence**	Social norm	0.28*	0.14	0.42**	0.42**															
	Social recognition	0.06	0.02	0.10	0.09	0.19														
	Social pressure	0.21	0.35**	0.32**	0.22	0.43**	0.48**													
**Identity/trait**	Risk-taking	0.37**	0.26*	0.24	0.16	0.15	0.27*	0.33*												
	Self-fulfilment	0.05	-0.07	0.19	0.06	0.30*	0.41**	0.46**	0.30*											
	Personal connection	0.10	0.05	-0.01	-0.06	0.13	0.18	0.10	0.35**	0.36**										
**Emotional attachment**	Positive emotions	0.04	-0.18	0.03	0.02	0.26*	0.26*	0.23	0.14	0.73**	0.30*									
	Negative emotions	0.01	-0.05	-0.13	-0.05	0.17	0.34**	0.28*	0.02	0.38**	-0.30	0.59**								
	Kin altruism	-0.03	-0.06	-0.15	0.01	0.07	0.07	0.06	0.29*	0.42**	0.31*	0.33**	0.15							
	Philanthropy	0.01	-0.02	-0.01	0.01	0.01	0.10	0.09	0.21	0.41**	0.18	0.29*	0.10	0.45**						
	Biophilia	0.12	-0.07	-0.04	0.02	0.14	0.15	0.02	0.22	0.38**	0.21	0.36**	0.20	0.57**	0.68**					
**Sustain-ability conscious-ness**	Concern	0.03	-0.10	-0.01	-0.10	0.04	0.24*	0.21	0.28*	0.49**	0.38**	0.37**	0.18	0.51**	0.54**	0.47**				
	Preference for sustainability	0.21	0.12	-0.06	0.00	0.14	0.42**	0.22	0.36**	0.39**	0.27*	0.33**	0.25*	0.34**	0.33**	0.30*	0.36**			
	Desire for having an impact	0.17	0.06	0.12	0.05	0.16	0.35**	0.33**	0.42**	0.57**	0.37**	0.48**	0.11	0.43**	0.44**	0.43**	0.64**	0.55**		
	Sense of responsibility	0.26*	0.21	0.13	-0.04	0.07	0.25*	0.32**	0.33**	0.49**	0.45**	0.36**	0.12	0.38**	0.47**	0.47**	0.66**	0.39**	0.54**	

(N = 68, * = *p* < 0.05, ** = *p* < 0.01, Likert-scale range from ‘*very influential* (7)’ motives to *’not influential at all* (1)’ motives, M = Mean, *SD* = Standard deviation, SE = Standard error).

Motives for making sustainable investments were widely similar between traditional and impact investors, and only marginal differences between the two types could be found (see Fig 2). Significant differences with large effect sizes between traditional and impact investors were only found in the two motives ‘preference for sustainability’ (*W* = 260, *p* < 0.01, Cohen’s *d* = 1.2) and ‘desire for wanting an impact’ (*W* = 381, *p* < 0.01, Cohen’s *d* = 1.3), both motives being more influential for impact investors than for traditional investors. Other significant differences between both investor types, however with moderate effect sizes, included ‘concern’ (*W* = 305 *p* < 0.01, Cohen’s *d* = 0.7), ‘sense of responsibility’ (*W* = 369, *p* < 0.01, Cohen’s *d* = 0.6), ‘philanthropy’ (*W* = 339, *p* < 0.01, Cohen’s *d* = 0.7), ‘kin altruism’ (*W* = 381, *p* < 0.05, Cohen’s *d* = 0.5) and ‘personal connection’ (*W* = 380.5, *p* < 0.05, Cohen’s *d* = 0.5). Subsequently, comparing both investor types at the level of motive-categories, the only significant difference was that impact investors had a stronger consciousness for sustainability that motivates them to invest sustainably (*W* = 200, *p* < 0.01, Cohen’s *d* = 1.19). Overall, motives for investing sustainably were found to be homogeneous across different socio-demographic and investor-based variables (e.g. gender and different age groups).

## Discussion

### Intention to invest in LDN

In this study, we aimed at explaining private-sector investors’ intention to invest in Land Degradation Neutrality (LDN) and analysing their motives for making investments that promote sustainable development. Regarding the actual intention to invest in LDN, the majority of investors showed rather weak intention to make investments that promote LDN in the near future. Interestingly, this weak intention to invest in LDN contrasts with investors’ relatively strong opinion on land degradation being a great danger to nature and humans, which also remains when comparing land degradation to other sustainable development goals (e.g. to end poverty or to promote peace). The combination of investors’ large concern about land degradation and the positive attitude towards LDN/investing sustainably with their weak intention to invest, can be seen as further evidence for the ‘sustainable investing gap’ [15] and investors’ general uncertainty regarding investment into natural resources (incl. ‘land’-based investments) [11].

In line with the main assumptions of the theory of planned behavior (TPB), we performed structural equation modelling (SEM) to empirically predict how investors’ intention to invest in LDN can be formed. Our SEM accounted for 53% of the variance in intention to invest in LDN, which can be considered relatively satisfactory, compared to the 39% explained variance in intention presented in the meta-analysis by Armitage & Conner (2001) [19]. Our SEM revealed that good knowledge and a positive attitude towards LDN/investing sustainably were strong predictors of intending to invest in LDN, e.g. in land restoration-promoting investments, provided that investors had sufficient financial resources and the general ability to invest sustainably. Subjective norm on the other hand was no significant predictor of the intention to invest.

Within the frame of this study, positive attitude reflected the investor’s belief that 1) LDN-based investments are a promising solution to combat land degradation and promote sustainable development in the long-term, 2) investing in sustainable investment products will yield both a high financial return and have a positive impact, and 3) investment products based on the LDN concept could be an attractive investment opportunity. Strong attitude-intention relationships have also been found in many other TPB-based studies [19] and are generally important for encouraging and integrating sustainability into behavior [28]. It is important to keep in mind that sustainable investments and especially LDN are relatively new investment opportunities, which might still be perceived as risky and uncertain. Nevertheless, we argue based on our SEM results that there is great potential in promoting a positive attitude towards LDN/investing sustainably in order to reinforce investors’ attitude-intention relationships.

Investors’ knowledge about LDN and investing sustainably was a good predictor of intending to invest in LDN. Yet there is a commonly found lack of knowledge and awareness about the cross-cutting and long-term consequences of land degradation, which is one of the key drivers of land degradation in the first place (e.g. in supply systems) [29,30]. Therefore, uncertainty and lack of knowledge about the causes and effects of land degradation could impede investors’ intention to invest in LDN and further prevent potential involvement. Addressing lack of knowledge and awareness about land degradation/LDN is essential, because investing sustainably is less likely the more barriers and uncertainty investors perceive between them and potential objects of investment [26]. Thus, spreading awareness and providing knowledge can be viewed as key factors for encouraging investors to invest in LDN.

In contrast with the assumptions of the TPB, investors’ intention to invest in LDN was not affected by perceived social norm (or even pressure) arising from their social networks and also not from public concern (’subjective norm’). Regarding social influence, special attention should be given to the role of public concern, which is found to be greatly influenced by the media [31]. Unlike climate change, which receives a high attention in the general media and therefore is, by now, a matter of wide public concern, land degradation is largely absent from the public debate. We hypothesise that if media coverage and, thus, public concern of land degradation was higher, the subjective norm might become predictive of investors’ intention to invest.

Furthermore, we found that knowledge had highly significant positive relationships with 1) impact investors, 2) investors who had closeness with the asset class ‘land’ (closeness-investors) and 3) investors who perceived themselves as victims of land degradation (victim-investors). We hypothesize that all three investor types (impact, closeness- and victim-investor) are already in a position which makes them more likely to acquire more knowledge about land degradation/LDN/investing sustainably than their counter-types (traditional investors, non-closeness and non-victims-investors). However, further research is needed to investigate this relationship.

Since intention was not directly predicted by being either a traditional or an impact investor, our results suggest that intention to invest in LDN is more influenced by psychological constructs that all investors commonly share, rather than by different type-specific investor characteristics.

### Motives for sustainable investing

Our results show that investors’ motivation to sustainably invest originates from multiple financial and non-financial influences, which adds further evidence for a behaviorally realistic amendment of the *homo economicus* concept [32]. Our results highlight the importance of psychological determinants (i.e. role of emotions and morals). The findings suggest that motives for investing sustainably were not only based on self-interest (e.g. financial return, portfolio diversification), but also on self-transcendence (e.g. concern about world’s future). This is in contrast with the belief that sustainable behavior can only be induced with the provision of proper economic incentives, such as monetary rewards or similar forms of compensation [33,34].

Average investor’s motivation for sustainable investments was found to be mainly triggered by motives of emotional attachment (i.e. love for people, nature and personal loved ones) and having established a consciousness for sustainability (i.e. concern, sense of responsibility, wanting to have an impact). These findings are supported in related studies highlighting the importance of emotional attachment as well as an ethical and moral value-belief-system for sustainable intentions and behaviors (here: consciousness for sustainability) [28,35,36]. In contrast, motives originating from social influence (e.g. following trends) were ranked by participants among the lowest motives when investing sustainably. This finding is in contrast with a general understanding that ‘herd behavior’ plays a crucial role in guiding individuals’ social and investor behaviour. [37,38]. Commonly expected motives of investors seeking to expand their wealth and influence (e.g. short-term financial return) were found to play a subordinate role in sustainable investing, as long as there was the prospect of financial returns in the long-term.

Surprisingly, traditional and impact investors seemed to roughly share the same motives when making sustainable investments. The only substantial differences were found in impact investors being more motivated by wanting to have an impact and having a preference for sustainability. Consequently, impact investors were found to have already established a stronger consciousness for sustainability. Interestingly, traditional and impact investors agreed on long-term financial returns being a relatively important motive for investing sustainably, whereas both found short-term financial returns to be less relevant in this regard. This finding contrasts with our expectation that private-sector investors’ generally focus on short-term financial returns, which is one of the most prominent barriers for investors engaging in investing sustainably (see the discussion of the ‘sustainable investing gap’ in [15]).

Despite the high variability in motives for sustainable investing across individual investors, we generally found low variability across different investor types, which is contrary to our prior expectations of discovering motivational patterns across different investor types (especially traditional vs. impact investors).

## Limitations

We used convenience sampling to recruit participants. Due to potential self-selection of respondents, this may have resulted in a non-representative the sample population.

Swiss investors (62%) made up the largest share of our sample. As Swiss public concern of environmental issues ranks higher than in other countries [39], generalising our findings may be restricted to Swiss individual investors. In addition, as sustainable investments are rapidly increasing in the Swiss market (up 169% in 2015; [40]), Swiss investors’ interest in sustainable investments may already be higher than that of investors from other countries, which could influence the outcomes of this study.

Most surveyed investors live in countries that are not the target of LDN investments. For them, an LDN investment would be an investment abroad, which may be subject to specific motivations and barriers. Our survey did not address such specific motivations for, or barriers against, investments abroad. Yet, they may have influenced the other responses and, hence, may have confounded our findings.

Also, due to lack of data, we could not include actual investments made in LDN, which therefore made it impossible to integrate actual behavior into our SEM. Even though theoretically assumed by the TPB, our model cannot guarantee that after forming the intention to invest in LDN, actual investments will be made. On the other hand, numerous studies in the empirical social sciences provide evidence for the crucial role of intention-forming for performing behavior [19].

Furthermore, as participants generally responded highly positive to the statements of the questionnaire, we cannot exclude the possibility of social desirability effect as well as answer bias. However, the SEM is based on relative, systematically differences between answers provided by individuals in the sample. We argue that if there was a strong bias due to socially desirable answers provided by participants, there would be little variance in the data, because of a bottom or ceiling effect. Too little variance in the data would mean that the model would not have fitted well, and that the paths coefficients could not be reliably estimated. Additionally, the respondents answered in general very positively to all statements in the questionnaire, which suggest that a possible bias would have affected all latent constructs in a comparable way. In other words, this would mainly affect the intercepts, but not the slopes of the estimated relations between the constructs in the SEM. We thus argue that it might be possible that manifest results could be affected by e.g., social desirability, but the possible bias is not so severe that the latent variables and the path coefficients in the SEM could not be reliably estimated.

Lastly, as we collected cross-sectional data, our analysis might not allow for strict causal interpretation of results.

## Conclusions

Our study suggests practical measures on how to best encourage private-sector investors to invest in LDN and, more generally, in sustainable development. This includes suggestions for how favourable circumstances can be created for investors intending to invest in emerging LDN financing vehicles:

- To encourage investors to make sustainable investments that promote LDN, the first step is to promote a clear and basic understanding about 1) the issue of land degradation, 2) how the adverse effects of land degradation might negatively affect private sector investors themselves and their businesses, 3) LDN as an effective solution to combat land degradation, 4) LDN as an investment opportunity with long-term expected return and a risk which is similar to that of other investments of similar time horizon, and 5) how investors can practically invest sustainably in LDN. Given the lack of knowledge and the high uncertainty that still surrounds the issue of land degradation, this requires above all improved and strengthened knowledge management, translation and transfer about land degradation itself.
- As the determinants of the intention and the motivation to invest sustainably do not substantially differ across different investor types, investors should be targeted as a uniform group. Emphasis should be placed on the psychological determinants they commonly share, rather than on the type-specific characteristics that may distinguish different investor types.
- That said, impact investors, investors who have natural resources (incl. land) as part of their investment strategy, and investors who perceive themselves as negatively affected by land degradation are still more likely to invest in LDN and thus are recommended as target groups.
- Attracting investors to engage in investing sustainably might strongly require the promotion of a consciousness for sustainability, for example by raising concern, evoking a sense of responsibility for future developments and highlighting the positive impact sustainable investments can have.
- Likewise, investors’ motivation to sustainably invest can be triggered by generating an emotional attachment to the investment object (e.g. stressing how the positive outcomes of investing sustainably would benefit their significant ones).
- In the context of sustainable investments, the prospect of long-term financial returns must be provided for investors, while short-term financial returns play a subordinate role.

## Supporting information

S1 File Questionnaire (incl. invitation letter for implicit consent). (PDF)

S2 File Survey data. (XLS)

S1 Table Items for motives for sustainable investing. (DOCX)
