# Supplementary material for: Private-sector investor’s intention and motivation to invest in Land Degradation Neutrality
Source: PLoS One. 2018 Dec 13;13(12):e0208813. doi: 10.1371/journal.pone.0208813 (PMC6292660; doi:10.1371/journal.pone.0208813)
Supplement: S1 File — (PDF) [file pone.0208813.s001.pdf]

University of Zurich[UZH]

Study: Motives for sustainable investments & Perception of land degradation

Part 1/2: Perception of land degradation

Dear investor,

In this study we will ask you questions about your motivation for making sustainable investments as well as your perception of land degradation and the concept of *Land Degradation Neutrality*.

Before answering the questions that follow, please read this short text

Land as an existential resource is being transformed and degraded due to global demands and human activities. This not only poses a major threat to the environment, but also impacts social welfare and food security of approximately one billion people. In response, the United Nations recognizes that combatting **land degradation** and restoring degraded land and soil are pivotal issues and therefore included them as objectives in the UN Agenda 2030 for Sustainable Development*.

**Sustainable development refers to societal/economic/environmental development to meet the needs of the present generation without compromising the ability of future generations to meet their needs.*

As land degradation due to unsustainable land use is estimated to cost about 490 billion USD per year, the finance sector will play a significant role in supporting the transition to sustainable land management. In this regard, the objective to avoid soil loss through the concept of **Land Degradation Neutrality (LDN)** is part of the UN Agenda to counteract land degradation.

The aim of LDN is to maintain and improve the amount of healthy and productive land resources over time by restoring 12 million hectares at an estimated cost of 2 billion USD per year. This represents the current land degradation footprint of the global economy and is a target to be achieved by 2030. LDN is a global initiative that primarily sees the solution in the promotion of sustainable land management techniques (e.g. agro-forestry) and land restoration/rehabilitation efforts including the generation of social and economic benefits for affected communities.

Investors can invest in **sustainable investment products** promoting LDN mainly through **green bonds** and **private equity impact investment funds** with the potential to halt and reverse the effects of land degradation.

Example of land degradation in Kongo 1979 vs. 2000. (Source: greenupgrader.com)

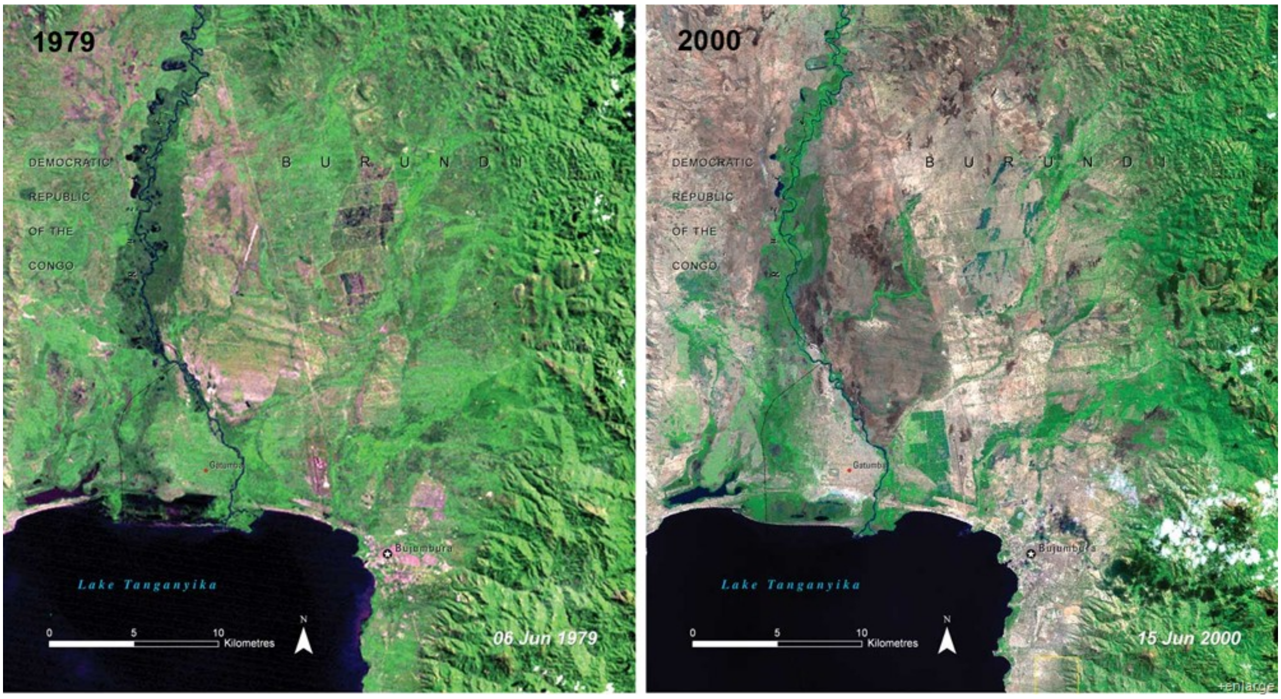

University of Zurich[UZH]

Study: Motives for sustainable investments & Perception of land degradation

Part 1/2: **Perception of land degradation**

*** 1. I am an ...**

◯ Independent investor ◯ Institutional investor

*** 2. How best would you describe your investment strategy?**

◯ Traditional investor: *I make investments primarily to generate financial returns.*

◯ Impact investor: *I make investments to generate financial returns that include having a positive social and/or environmental impact.*

◯ Philanthropic investor: *I make investments primarily to generate a positive social and/or environmental impact and less/no financial returns for myself.*

*** 3. Are your investment decisions independent of restrictions set by somebody else (e.g. by your employer, your clients, etc.)?**

◯ Independent ◯ It varies ◯ Not independent

*** 4. Are natural resources (including 'land') as an asset class part of your investment strategy?**

◯ Yes ◯ No ◯ Maybe

5. Only if 'NO' for question 4, please answer this question: Why do you not include natural resources (including 'land') as part of your investment strategy?

◯ High uncertainty ◯ Low expected return ◯ No specific reason

Other (please specify)

[ ]

6. Only if 'YES' for question 4, please answer this question: In the past FIVE years, have you already made at least ONE investment in sustainable land management, land rehabilitation, restoration AND/OR similar conservation purposes?

◯ Yes ◯ No ◯ Maybe

*** 7. Please rate the following statements.**

	Strongly agree	Agree	Somewhat agree	Somewhat disagree	Disagree	Strongly disagree
I think land degradation is a great danger to nature and humans.	○	○	○	○	○	○
Compared to other objectives within the UN Sustainable Development Goals (e.g. mitigate climate change, promote peace or end poverty) land degradation is something I consider a serious threat to humans and the environment.	○	○	○	○	○	○
To the best of my knowledge, I think *Land Degradation Neutrality* is a promising solution to counteract land degradation in the long-term.	○	○	○	○	○	○
I think sustainable investment products based on the concept of *Land Degradation Neutrality* could be an attractive investment opportunity.	○	○	○	○	○	○
Generally, I am confident that investing in sustainable investment products will yield a high financial return and have a positive impact.	○	○	○	○	○	○
I am convinced that sustainable investment products promote sustainable development in an effective way.	○	○	○	○	○	○
The people in my working life, whose opinion I value, expect me to invest in *Land Degradation Neutrality* in an effort to combat land degradation.	○	○	○	○	○	○
The people in my private life, whose opinion I value, expect me to invest in *Land Degradation Neutrality* in an effort to combat land degradation.	○	○	○	○	○	○
There is strong public concern about land degradation as a serious threat, which is one of the reasons, I should make investments in *Land Degradation Neutrality* in an effort to combat it.	○	○	○	○	○	○
I am NOT in a position to invest in *Land Degradation Neutrality* because I do NOT know how to do so.	○	○	○	○	○	○

	Strongly agree	Agree	Somewhat agree	Somewhat disagree	Disagree	Strongly disagree
I have the ability/financial resources to invest in sustainable development AND/OR *Land Degradation Neutrality.*	◯	◯	◯	◯	◯	◯
I will invest/increase my investments in *Land Degradation Neutrality* in 2016/2017 to make a contribution to counteracting land degradation.	◯	◯	◯	◯	◯	◯

*** 8. I am directly affected by the negative effects of land degradation.**

◯ Yes ◯ No ◯ Maybe

*** 9. My previous investment decisions may have directly contributed to generating land degradation.**

◯ Yes ◯ No ◯ Maybe

University of Zurich[UZH]

Study: Motives for sustainable investments & Perception of land degradation

Part 2/2: **Motives for sustainable investments**

Instruction: Please rate each of these statements ('motives'). **How strongly does the motive given influence your decision-making process to invest in products promoting sustainable development?**

If your investment decision-making is not independent of constraints set by somebody else, please hypothetically assess each reason as an independent decision-maker.

* **10. Generally, I make investments that promote sustainable development, because...**

	Very influential (7)	(6)	(5)	(4)	(3)	(2)	not influential at all (1)
... of an expected high financial return (long-term).	○	○	○	○	○	○	○
... of an expected high financial return (short-term).	○	○	○	○	○	○	○
... I feel responsible for the world's future.	○	○	○	○	○	○	○
... sustainable investments are trendy and I do not want to miss out on promising prospects of a growing market.	○	○	○	○	○	○	○
... it makes me happy/feel positive.	○	○	○	○	○	○	○
... I feel guilty about my/our wrongdoing of unsustainable behaviour/investments.	○	○	○	○	○	○	○
... I generally endorse anything sustainable and therefore choose sustainable investments over other investment opportunities.	○	○	○	○	○	○	○
... it benefits my image/reputation.	○	○	○	○	○	○	○
... I want to diversify my portfolio.	○	○	○	○	○	○	○
... people close to me support the idea of me making sustainable investments.	○	○	○	○	○	○	○
... it is personally fulfilling.	○	○	○	○	○	○	○

	Very influential (7)	(6)	(5)	(4)	(3)	(2)	not influential at all (1)
... I am concerned about the issues our planet/society will have to face in the future because of our current unsustainable behaviour.	◯	◯	◯	◯	◯	◯	◯
... I have a personal connection to the object of my investment.	◯	◯	◯	◯	◯	◯	◯
... I want to ensure the future safety/well-being of my loved ones (e.g. family).	◯	◯	◯	◯	◯	◯	◯
... of my general love of humans, who will benefit if I do.	◯	◯	◯	◯	◯	◯	◯
... of my general love of nature (animals/plants/ecosystems), which will benefit if I do.	◯	◯	◯	◯	◯	◯	◯
... of the expected gain of subsidies, tax benefits AND/OR market-based incentives.	◯	◯	◯	◯	◯	◯	◯
... even if there may be some risks, I like to take risks.	◯	◯	◯	◯	◯	◯	◯
... I want to have a positive impact.	◯	◯	◯	◯	◯	◯	◯

Other (please specify)

[ ]

*** 11. I was born in... (year)**

[ ]

*** 12. I am...**

◯ Male ◯ Female

*** 13. I am currently living in... (country)**

[ ]

14. (optional): Thank you for your participation. Do you have any comments?

[ ]

15. (optional): If you are interested in the results of this study, please leave your email address.

[ ]

Study invitation

Subject: **Motives for sustainable investments & Land Degradation Neutrality - your participation is important!**

Dear Mr / Ms ...,

Our future environment will be shaped through individual beliefs and behaviour. Therein, investors and their decision-making towards allocation of financial resources into sustainable investments play a crucial role, which raised the questions for this study:

- Why do people invest money into a sustainable future?
- How do motives and perceptions influence sustainable investor behaviour?
- Understanding the motives and perceptions for different types of investors, how could this knowledge support sustainable development?

In addition, this study explores investor's perception of land degradation as an environmental issue and the newly-emerging concept of *Land Degradation Neutrality* as a potential solution to halt and reverse land degradation.

We cordially invite you to participate in this scientific study by following the link to the online survey (total estimated time **10 minutes**):

https://de.surveymonkey.com/r/motivesforsustainableinvestorbehaviour

Your answers will be used for the sole purpose of this scientific study and will not be used commercially or shared with third parties.

In the attachment you will find the detailed research outline for this study. If you send us your email address separately or add it to the online-survey, it will be our pleasure to inform you about the outcomes of this study by December 2016.

If you have any questions or concerns, please contact: Mr Tony Reyhanloo, tonyshahin.reyhanloo@uzh.ch

Thank you very much for your valuable contribution.

With best regards,

Tony Reyhanloo and Philippe Saner, PhD
Department of Evolutionary Biology and Environmental Sciences, University of Zurich
Winterthurerstrasse 190, CH-8057 Zurich
Switzerland

Universität Zürich[UZH]

Contact: **Tony Shahin Reyhanloo**
tonyshahin.reyhanloo@uzh.ch
at the Department of Evolutionary Biology and
Environmental Studies (DEBES)

Information sheet on the study
Motives for sustainable investor behaviour
- in the context of land degradation -

Research interest: Why do people invest money into sustainable development? What are the underlying motives of such sustainable behaviour? By combining these questions to an environmental problem that has not gained as much public attention as other issues (e.g. climate change), this interdisciplinary study focuses on sustainable investor behaviour and the perception of *Land Degradation Neutrality* as a promising solution to halt and reverse land degradation.

Importance of study: To what extent sustainable investor behaviour is triggered by motives is unknown and so is their potential power of influencing individual behaviour and thus financial flows. This counts likewise for general perception of land degradation as a challenge to sustainable development. There is also little knowledge on the impact investments from the private sector can have for personal and overall environmental and humanitarian gain. Therefore, understanding motives and perceptions of individuals can lead to new insights how individual behaviour and investments can shape our future landscape.

Participants: **Investors**

Study procedure: The completion of the **online questionnaire** will only take about **10 minutes**. Data acquisition will be processed **strictly confidential** and **anonymously evaluated** as well guaranteed only to be used for the purposes of this scientific study. Please follow this link:

➔ https://de.surveymonkey.com/r/motivesforsustainableinvestorbehaviour 🡐

Compensation for study participation: To thank you for participating we will send you the anonymised overall results of the study later this year. By participating you will make a great contribution reaching the overarching goals of this study:

- generating a better understanding of the perception of land degradation and Land Degradation Neutrality
- analysing motives for sustainable investor behaviour,
- creating profiles for different investor groups (traditional, impact & philanthropic type),
- enabling innovative solutions how to use that knowledge in order to increase sustainable investor behaviour and thus sustainable development.

If you have questions regarding the study, please do not hesitate to contact us.

Thank you!
