# Supplementary material for: Private-sector investor’s intention and motivation to invest in Land Degradation Neutrality
Source: PLoS One. 2018 Dec 13;13(12):e0208813. doi: 10.1371/journal.pone.0208813 (PMC6292660; doi:10.1371/journal.pone.0208813)
Supplement: S1 Table — (DOCX) [file pone.0208813.s003.docx]

S1 Table - Items for Motives for sustainable investing

| **Presented statement in questionnaire:**  *"Generally, I make investments that promote sustainable development, because..."* | **Motive-Category** | **Motive** | **Definable characteristics**  (SI = Sustainable investing) |
| --- | --- | --- | --- |
| ... of an expected high financial return (long-term). | **WEALTH GAIN** | **High financial return**  **(long-term)** | Motive for SI triggered by wanting to maximize/ensure wealth/influence (long-term) through positive financial performance. |
| ... of an expected high financial return (short-term). |  | **High financial return**  **(short-term)** | Motive for SI triggered by wanting to maximize/ensure wealth/influence (short-term) through positive financial performance. |
| ... I want to diversify my portfolio. |  | **Portfolio diversification** | Motive for SI triggered by wanting to diversify investment portfolio with the intention of minimizing exposure of asset classes to risk and/or to have a need for innovation, which both eventually maximise/ensure position of influence/wealth. |
| ... of the expected gain of subsidies, tax benefits AND/OR market-based incentives. |  | **Incentives** | Motive for SI triggered by wanting to receive both regulatory and market-based incentives (e.g. subsidies or tax benefits), and therefore maximize wealth/influence. |
| ... sustainable investments are trendy and I do not want to miss out on promising prospects of a growing market. | **SOCIAL INFLUENCE** | **Social norm ('affiliation to trend')** | Motive for SI triggered by wanting to follow the expectations of an economic/social trend (here: growing market) with the promising prospect of expanding position of wealth in doing so; at the same time this is influenced by social pressure and affiliation to the trend of not wanting to miss out. |
| ... it benefits my image/reputation. |  | **Desire for social recognition** | Motive for SI triggered by wanting to enhance public and professional self-portrayal (includes image, reputation, transparency), which eventually increases attractiveness for future social interactions and businesses. |
| ... people close to me support the idea of me making sustainable investments. |  | **Social pressure** | Motive for SI triggered by direct or indirect social pressure on the individual's emotions, opinions and/or behaviour being affected as product of social interactions. Sub-forms of social pressure can be psychological phenomena such as conformity, social cohesiveness, social desirability, reciprocity, persuasion and the like. |
| ... even if there may be some risks, I like to take risks. | **IDENTITY/ TRAIT** (RESIDUAL GROUP) | **Risk-taking trait** | Motives for SI triggered by individual character trait of risk-taking (associated with need for exploration/simulation/excitement/curiosity/ openness/sensation-seeking). |
| ... it is personally fulfilling. |  | **Self-fulfilment** | Motive for SI triggered by wanting to enhance/fulfill self-ideal through interaction with object of interest and the impact it may have in return. |
| ... I have a personal connection to the object of my investment. |  | **Personal connection** | Motive for SI triggered by having a personal connection to object of investment, which can be objective and/or subjective (e.g. origin of birth vs. liking of a particular country). |
| ... it makes me happy/feel positive. | **EMOTIONAL ATTACHMENT** | **Positive emotions** | Motive for SI triggered by anticipative or subsequent feelings of positive emotions (satisfaction, joy and happiness) through the act of investing sustainably. |
| ... I feel guilty about my/our wrongdoing of unsustainable behaviour/  investments. |  | **Negative emotions** | Motive for SI triggered by feelings evoking negative emotions (e.g. remorse, guilt, bad conscience, indignation, fear), which are a consequence of past behaviour that was personally evaluated as non-sustainable ('negative') and thus motivate future sustainable-behaviour. |
| ... I want to ensure the future safety/well-being of my loved ones (e.g. family). |  | **Kin altruism** | Motive for SI triggered by the wanting to secure personal and/or loved one's vulnerability/well-being/health/existence, includes strong interpersonal relationships (such as family and close/distant relatives with strong bonds). |
| ... of my general love of humans, who will benefit if I do. |  | **Philanthropy** | Motive for SI triggered by feeling goodwill/love/appreciation for people and being emotionally connected to the present and future welfare of people/the human race. |
| ... of my general love of nature (animals/plants/ecosystems), which will benefit if I do. |  | **Biophilia** | Motive for SI triggered by feeling goodwill/love/appreciation for nature and being emotionally connected to nature and caring about the further development of natural systems. |
| ... I am concerned about the issues our planet/society will have to face in the future because of our current unsustainable behaviour. | **SUSTAINABILITY CONSCIOUSNESS** | **Concern** (surrounding 'sustainable issues') | Motive for SI triggered by concern (includes awareness/affinity) to issues surrounding sustainable development (includes three pillars of economy, environment and society), which has a guiding force in doing so. |
| ... I generally endorse anything sustainable and therefore choose sustainable investments over other investment opportunities. |  | **Preference for sustainability** | Motive for SI triggered by individual preference (inherently being in favour of something/liking something over something else) and positive attitude, which in addition can become a relatively automatic cognitive decision-making process and a form of habituation over time (e.g. habit of making investments that promote sustainable development). |
| ... I want to have a positive impact. |  | **Desire for having an impact** on a sustainable future | Motive for SI triggered by desire for having a positive impact ('giving back') by promoting sustainable development (includes all three pillars: society/environment/economy) in any shape or form. Altruism as an example can be an adequate form of it through acts of selflessness, but not a mandatory one. |
| ... I feel responsible for the world's future. |  | **Sense of responsibility** for a sustainable future | Motive for SI triggered by feeling a driving sense of responsibility/moral and ethical obligation due to identified impact of personal/societal actions of present on future development. |
